# KIM-1: A Non-Invasive Marker for Kidney Injury and Treatment Efficacy in FMF

**DOI:** 10.31138/mjr.210625.are

**Published:** 2026-06-28

**Authors:** Oktay Bagdatoglu, Esra Fırat Oguz, Ahmet Kor, Sukran Erten, Mıne Sebnem Karakan

**Affiliations:** 1 Department of Nephrology Clinic, Republic of Turkey Ministry of Health Ankara Bilkent City Hospital, Ankara, Türkiye;; 2 Department of Biochemistry Clinic, Republic of Turkey Ministry of Health Ankara Bilkent City Hospital, Ankara, Türkiye;; 3 Department of Rheumatology Clinic, Republic of Turkey Ministry of Health Aksaray Training and Research Hospital, Aksaray, Türkiye;; 4 Department of Rheumatology Clinic, Ankara Yıldırım Beyazıt University-Ankara Bilkent City Hospital, Ankara, Türkiye,; 5 Department of Nephrology, Ankara Yıldırım Beyazıt University – Ankara Bilkent City Hospital, Ankara, Türkiye

**Keywords:** Kidney Injury Molecule 1, KIM-1, Familial Mediterranean Fever, canakinumab, GFR

## Abstract

**Objective:**

Familial Mediterranean Fever (FMF) arises from mutations in the MEFV gene, which encodes the immune-regulatory protein pyrin. Early detection of FMF-associated kidney injury and identification of noninvasive biomarkers to monitor disease progression and treatment response are critical. This study aimed to measure serum Kidney Injury Molecule-1 (sKIM-1) levels in FMF patients, assess its potential as an early indicator of renal damage, and evaluate its utility in monitoring treatment efficacy.

**Methods:**

Fifty FMF patients and 36 healthy controls were enrolled. Serum KIM-1 levels were determined by ELISA. Clinical and laboratory data were extracted from patient records. The relationships between sKIM-1, urine protein/creatinine ratio (UPCR), and estimated glomerular filtration rate (eGFR) were analyzed. Subgroup analyses compared sKIM-1 levels in patients treated with anakinra or canakinumab.

**Results:**

Median sKIM-1 was significantly higher in FMF patients than controls [571.30 pg/mL (562.50–586.90) vs. 562.40 pg/mL (558.10–580.40), p=0.040]. No correlation was observed between sKIM-1 and UPCR (p=0.547) or eGFR (p=0.232) in the FMF group. Canakinumab-treated patients exhibited significantly lower sKIM-1 levels [561.10 pg/mL (556.10–567.00) vs. 575.4 pg/mL (564.00–591.20), p=0.036], whereas anakinra treatment showed no significant difference [561.20 (558.40–595.70), 572 (564.60–588), (p=0.430)].

**Conclusion:**

Elevated sKIM-1 in FMF patients and its reduction with canakinumab support KIM-1 as a noninvasive biomarker of renal involvement in FMF. These findings suggest KIM-1 may serve as a tool for monitoring disease activity and treatment response.

## INTRODUCTION

Familial Mediterranean Fever (FMF) is a hereditary autoinflammatory disease, particularly affecting populations of Mediterranean origin, and it is the most common inherited autoinflammatory disease worldwide.^1^ In Turkey, the prevalence of FMF is generally reported to be 1/1000.^2^ Renal amyloidosis associated with FMF is the most significant complication, which can lead to proteinuria and subsequently renal failure.^1^

KIM-1, both a kidney and liver-expressed type 1 trans-membrane protein.^3^ It consists of two parts: extracellular and cytoplasmic. The extracellular part contains six cysteine immunoglobulin-like domain and a Thr/ Ser-Pro-rich domain with mucin-like O-glycosylated proteins.^4^ The cytoplasmic domain comprises KIM-1a and KIM-1b variants. While KIM-1a is synthesised by hepatocytes, the KIM-1b variant is mainly synthesised by the kidneys. KIM-1 is also known as T cell immunoglobulin mucin domain-1 (TIM-1), which is a shared stimulatory molecule of T cells capable of enhancing T cell proliferation and cytokine production.^5,6^

In patients with FMF, Kidney Injury Molecule-1 (KIM-1) detects early and subclinical tubular injury with greater sensitivity than classical renal biomarkers such as serum creatinine, eGFR, and UPCR.^7^ KIM-1 expression reflects ongoing damage in the renal cortex and tubulointerstitial compartments; even in cases of mild AKI, serum KIM-1 levels rise before histopathological changes become apparent.^7–9^

Proinflammatory cytokines in FMF (e.g., IL-1β, TNF-α) can induce oxidative stress and tubular injury, which may evade detection by conventional parameters, manifesting instead as isolated elevations in KIM-1.^10^ Although biomarkers such as uromodulin provide valuable information for assessing overall kidney function and CKD risk, their sensitivity for detecting early tubular injury in FMF appears somewhat lower compared to KIM-1.^11–13^ Therefore, this study aimed to evaluate the potential diagnostic value of KIM-1 for monitoring subclinical or pre-amyloid renal damage and therapeutic response in FMF. Because of its specificity for tubular injury in glomerular and interstitial lesions—particularly in AA amyloidosis—KIM-1 was adopted as the primary biomarker for early-stage injury detection rather than as a complementary marker.^14,15^

When assessing kidney function, biomarkers other than serum urea nitrogen, creatinine, and estimated glomerular filtration rate can also be utilised. KIM-1 can provide insight into the prognosis of kidney damage.^16,17^ Studies showing that cells expressing KIM-1 produce more chemokines or cytokines under hypoxic conditions have demonstrated the association of KIM-1 with inflammation and fibrosis.^18,19^ Previously, sKIM-1 levels have not been studied in the FMF patient group, and the relationship between sKIM-1 and agents used in FMF or genetic variations in FMF is unknown.

In this study, we attempted to examine the relationship between KIM-1 indicating kidney damage and FMF, an autoinflammatory hereditary disease. For this purpose, we compared sKIM-1 levels with FMF patients and healthy control groups and investigated the relationship between s-KIM-1 and agents used in FMF treatment and genetic variations in FMF.

## MATERIALS AND METHODS

Between December 2023 and February 2024, a total of 50 participants diagnosed with FMF based on genetic mutations and clinical parameters, aged 18 or older, were randomly selected from outpatient clinics of the Nephrology and Rheumatology Departments. Additionally, 36 healthy participants were included in the control group. The control group was selected to have similar age, gender, body mass index, smoking status, and comorbid diseases with the FMF group. People with any rheumatic or renal disease were not included in the control group, and people with any rheumatic or renal disease other than FMF were not included in the FMF group. All FMF patients met the Tel-Hashomer criteria.^20^ In the patient group, there were 35 male and 15 female patients, while in the control group, there were 27 male and 9 female participants. Patient data were obtained from hospital records, and sKIM-1 levels were measured from leftover serum samples collected for routine laboratory parameters. Among the patient group, 45 individuals were using colchicine, while 5 were not.

The estimated glomerular filtration rate (eGFR) was calculated using the CKD-EPI 2009 Formula (for patients aged 18 and older).^21^ Our study, approved by the hospital’s ethics committee, was designed in accordance with the Helsinki Declaration. Informed consent was obtained from participants in both the patient and control groups.

### Biochemical Analyses

Venous blood samples were collected into vacutainer tubes and centrifuged at 1300 × g for 10 minutes. Separated sera were stored at −80 °C until analysis. Serum urea, creatinine (sCr), albumin (sAlb), C-reactive protein (CRP), spot urine protein and spot urine creatinine levels were measured at Atellica Solutions autoanalyser (Siemens Healthineers, Mannheim, Germany). Estimated glomerular filtration rate (eGFR) was calculated according to the CKD-EPI 2009 formula.

Serum Human Kidney Injury Molecule 1 (KIM 1) levels were measured with a commercially available ELISA kit (USCN, Wuhan, China) using the quantitative sandwich enzyme immunoassay technique. The detection range of the KIM 1 assay (catalog no:SEA785Hu-lotno:L230216193) was 78–5000 pg/mL and the sensitivity of the assay was 32 pg/mL. Intra- and interassay precision were <10% and <12%, consecutively.

### Statistical Analysis

To determine the normal distribution of continuous variables, the Shapiro-Wilk Test test and Q-Q plots, Box plots, and histogram graphics were utilised. Descriptive statistics were presented as mean and standard deviation (mean±SD) for normally distributed variables, while median (interquartile range [IQR], [25%–75%]) was used for variables with non-normal distribution. Additionally, two-sided comparisons between groups were evaluated using Independent Samples T-Test for normally distributed variables and Mann-Whitney U test for non-normally distributed variables. Correlation analyses between study parameters were conducted using Spearman and Pearson correlation analyses (R-values: 0.2–0.4 mild correlation; 0.4–0.8 moderate correlation; 0.8–1.0 high correlation). For comparisons among multiple groups, One-Way ANOVA post hoc Tukey test was used for normally distributed quantitative variables, while Independent Samples Kruskal-Wallis test was used for non-normally distributed quantitative variables. Chi-square and Fisher’s exact tests were employed for comparing categorical data. Values at the level of <0.05 were accepted as the significant limit for P value. Statistical analyses were performed using the Statistical Packages for the Social Sciences (SPSS) version 22.0 software package.

## RESULTS

**Figure 1.**
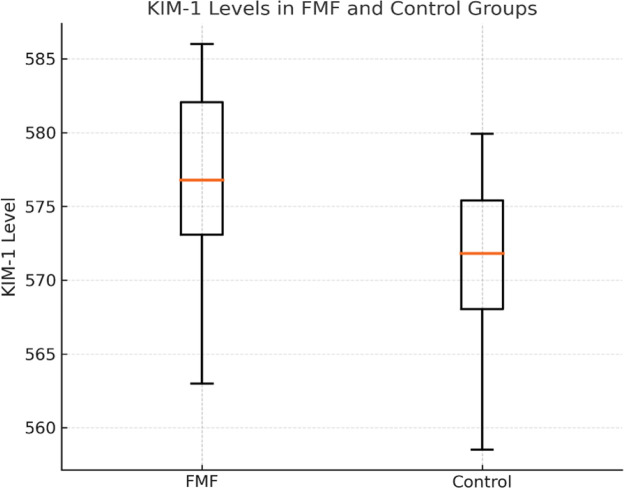
KIM-1 levels in patients with Familial Mediterranean Fever (FMF) and healthy controls. Box plots display the median and interquartile ranges (IQR). Whiskers indicate the range excluding outliers. KIM-1 levels were significantly higher in the FMF group compared to controls (p = 0.040).

Comparison of demographic characteristics and laboratory data between groups is presented (**Table 1**). In pairwise comparisons, age, gender, smoking status and presence of comorbid diseases were found to be similar between the groups (p>0.05). While the median values of glucose, urea, creatinine, sodium, potassium, phosphorus, albumin and CRP were similar between the groups, the median value of KIM-1 was found to be significantly higher in the FMF group compared to the controls [571.3 (562.5–586.9), (562.4 (558.1–580.4), p: 0.04)]. **Figure 1** shows the box-plot graph of the KIM-1 distribution among groups.

**Table 1.** Comparison of demographic characteristics and laboratory data between groups.

	FMF	CONTROL	P
Number of Participants	50	36	
Gender Female/Male, n	35/15	27/9	0.813
Age, Mean ± SD (Years)	41.60 ±11.80	47.90 ±13.40	0.162
Body Mass Index, Mean ± SD (kg/m^2^)	26.50±4.53	27.80±6.21	0.461
Presence of Comorbid Disease			
Diabetes Mellitus Hypertension Chronic Obstructive Pulmonary Disease Coronary Artery Disease	3 5 2 5	2 3 2 1	0.626 0.519 0.582 0.111
Smoking, n (%)	3	4	0.348
Glucose, Mean ± Sd (mg/dL)	82.50 (73.70–90.20)	84 (80.50–92.80)	0.167
Creatinine, Mean ± Sd (mg/dL)	0.75 (0,70–9)	0.7 (0.70–0.80)	0.499
Urea, Median (IQR) (mg/dL)	27.90 (19.20–33.07)	30.20 (23.60–34.80)	0.119
eGFR, Median (IQR) (mL/min/1.73 m^2^)	105.50 (93.70–121)	100 (91.50–115)	0.232
Sodium, Median (IQR) (mmol/L)	139 (102–140.70)	137 (100.60–140.40)	0.347
Potassium, Median (IQR) (mmol/L)	4.10 (3.90–4.40)	3.80 (3.70–4.20)	0.139
Phosphorus, Median (IQR) (mg/dL)	3.20 (2.60–3.90)	3.40 (2.70–3.60)	0.510
Albumin Median (IQR) (g/L)	44.70 (42.60–47.10)	44.90 (42.70–47.60)	0.850
CRP, Median (IQR) (mg/L)	2.05 (0,50–10.60)	1.90 (0,70–5.30)	0.547
UPCR, Median (IQR) (mg/g)	92.40 (61.40–139.60)	52 (44.70–84.30)	0.103
KIM-1, Median (IQR) (pg/mL)	571.30 (562.50–586.90)	562.40 (558.10–580.40)	0.040

CRP: C- Reactive Protein; eGFR: Estimated Glomerular Filtration Rate; UPCR: Urine Protein Creatinine Ratio; KIM 1: Kidney Injury Molecule 1.

**Figure 2.**
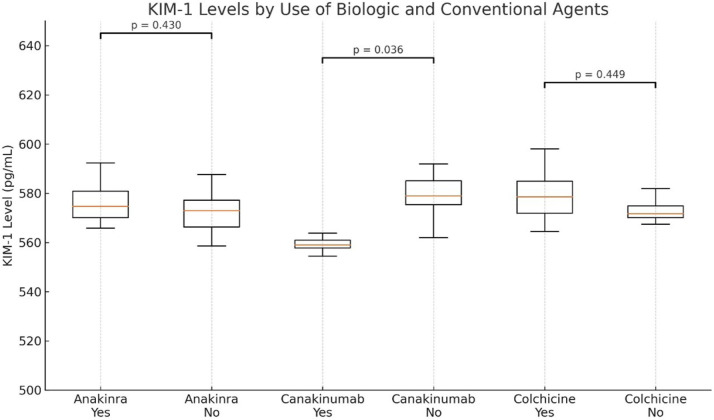
Box plot representation of KIM-1 levels in FMF patients according to Anakinra, Canakinumab, and Colchicine use. Values are shown as medians with interquartile ranges. Statistically significant differences were tested between users and non-users of each agent. A significant difference was observed in KIM-1 levels between Canakinumab users and non-users (p = 0.036). No significant differences were detected for Anakinra (p = 0.430) or Colchicine (p = 0.449).

**Table 2** illustrates the relationship between certain clinical conditions and medications used in medical treatment with KIM-1 in the FMF group. Current FMF attacks, genetic mutations, frequency of FMF attacks, presence of peripheral arthritis, colchicine use and dosage, anakinra, and canakinumab usage were identified from the patient group’s medical records. Statistically significant lower sKIM-1 levels were observed in patients using canakinumab in the FMF patient group [respectively, 561.10 (556.10–567), 575.4 (564–591.20), p=0.036] Although a lower level of KIM-1 was found in patients using anakinra, no statistical significance was detected [561.20 (558.40–595.70), 572 (564.60–588), (p=0.430)]. There was no statistically significant relationship between other laboratory and demographic data of the patient and control groups and sKIM-1 (**Table 2**). **Figure 2** shows the box-plot graph of the distribution of KIM-1 in subgroups created according to drug use in FMF.

**Table 2.** The relationship between some clinical conditions and KIM-1 in the FMF group.

		n	KIM-1 level Median (IQR)	P-value
Current FMF Attack	Yes	10	579.80 (563.60–641.50)	0.297
	No	40	568.90 (561.80–583.70)	
Gene Mutation	M694V MAN	12	572 (558.70–597.20)	0.487
	M694V HOT	20	567.70 (563.70–585.40)	
	M694V/M680I COMPOUND HE	4	561.90 (557.70–587.10)	
	Other Mutation	10	583 (569.40–602.90)	
	Negative	4	569.10 (557.40–624.20)	
Peripheral Arthritis	Yes	12	573.60 (561.80–587.60)	0.874
	No	38	570.90 (563.60–588)	
Use of Colchicine	Yes	45	573.50 (562.20–588)	0.449
	No	5	566.10 (562.50–580.90)	
Colchicine Requirement (Day/0.5 mg Tb)	0	5	561.50 (557.10–576.20)	0.942
	1	2	558.20 (567.30-...)	
	2	21	577.30 (566.2–5830)	
	3	16	569.20 (561.80–593.40)	
	4	6	568.40 (556.60–278.10)	
Use of Anakinra	Yes	5	561.20 (558.40–595.70)	0.430
	No	45	572 (564.60–588)	
Use of Canakinumab	Yes	4	561.10 (556.10–567)	0.036*
	No	46	575.40 (564–591.20)	

**Table 3** presents the individual clinical and laboratory data of FMF patients treated with canakinumab. Notably, serum KIM-1 levels among canakinumab-treated FMF patients remained tightly clustered between 556.10 and 567.30 pg/mL, despite variability in UPCR and eGFR values.

**Table 3.** Individual clinical and laboratory data of FMF patients using canakinumab.

Patient ID	Age (years)	Sex	UPCR (mg/g)	eGFR (mL/ min/1.73m^2^)	KIM-1 (pg/mL)
Patient 1	20	Male	111.00	101	556.20
Patient 2	26	Male	87.90	125	566.10
Patient 3	45	Female	179.00	67	556.10
Patient 4	22	Female	81.50	110	567.30

FMF: Familial Mediterranean Fever; UPCR: Urine Protein-to-Creatinine Ratio; eGFR: Estimated Glomerular Filtration Rate; sKIM-1: serum Kidney Injury Molecule-1.

**Figure 3.**
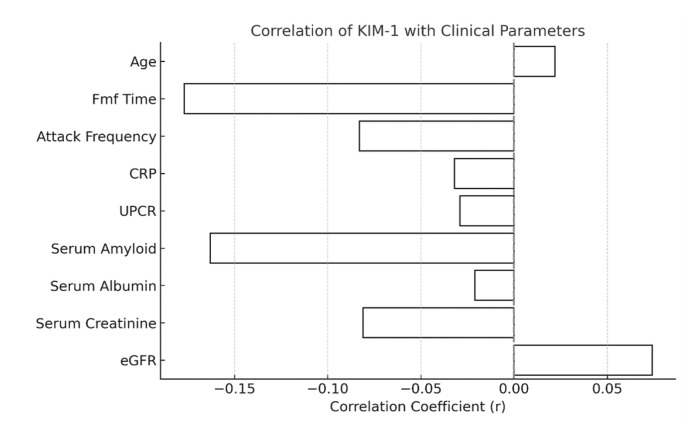
Correlation of KIM-1 levels with various clinical parameters. Bars represent Pearson correlation coefficients (r). Positive and negative correlations are shown relative to the zero reference line. All associations were statistically non-significant (p > 0.05).

The correlation relationship between certain study parameters and KIM-1 in the FMF group is demonstrated in **Figure 3**. In the FMF patient group, no statistically significant correlation was found between age, duration of FMF, frequency of FMF attacks, UPCR, serum amyloid, serum albumin, serum creatinine, and eGFR values with sKIM-1 levels.

## DISCUSSION

FMF occurs with the development of mutations in the gene (MEFV), which encodes the protein called pyrin, having regulatory functions on the immune system.^22^ Most FMF-associated mutations are located in exons 2, 3, 5, and 10. The most common mutations are M694V, M680I, V726A, M694I in exon 10 and E148Q in exon 2. These mutations account for approximately two-thirds of classic cases.^23^

FMF attacks typically begin in early childhood, and 80–90% of patients become symptomatic before the age of 20.^1^ FMF presents with recurrent serositis (peritonitis, pleuritis, arthritis) and fever attacks. Fever and peritonitis are the most commonly encountered symptoms, seen in over 90% of patients.^24,25^

Mutations in the MEFV gene disrupt the interaction of pyrin with microtubules, PKN, and 14-3-3 proteins, facilitating the formation of a proinflammatory pyrin inflammasome. When the pyrin inflammasome assembles, it activates caspase-1, leading to the formation of mature forms of pro-IL-1β and pro-IL-18, namely IL-1β and IL-18, which in turn triggers cell death known as pyroptosis. The excessive activation of the pyrin inflammasome and subsequent inflammation trigger the typical febrile attacks observed in FMF.^26,27^ Due to the relationship between FMF and chronic inflammation, colchicine and interleukin (IL)-1 antagonists are widely used in the treatment of FMF. Although genetic tests are used in the diagnosis of FMF, there is a need to develop biomarkers to assess the severity, activity, and response to treatment of FMF. Therefore, the use of serum or urine biomarkers in patients who cannot undergo kidney biopsy may be beneficial.

KIM-1, a phosphatidylserine receptor that confers a phagocytic phenotype to renal epithelial cells. It can specifically recognise phosphatidylserine epitopes on the surface of apoptotic tubular epithelial cells. Consequently, apoptotic and necrotic material resulting from injury can be phagocytosed from the tubular lumen through a combination of KIM-1 and phosphatidylserine epitopes.^28^ Additionally, it has been shown that the upregulation of KIM-1 expression is associated with the differentiation and proliferation of cells in damaged areas to form functional epithelium.^3^

The activation of the G protein α12 (Gα12) subunit by reactive oxygen species is the main cause of tissue damage during renal ischemia-reperfusion injury.^29^ KIM-1 inhibits the activation of Gα12 by blocking the binding of GTP to Ga12.^30^ KIM-1 may provide protection against Ga12-mediated tissue damage during ischemic acute kidney injury. However, it is believed that the inhibition of Ga12 by KIM-1 is temporary.^31^

Van Timmeren et al. demonstrated that positive KIM-1 staining in undifferentiated proximal tubular cells in tissue samples obtained from 102 patients undergoing kidney biopsy for various kidney diseases correlated with tubulointerstitial fibrosis and inflammation. In a subset of patients who underwent urine collection procedure close to the time of biopsy, urinary KIM-1 levels correlated with tissue expression of KIM-1.^32,33^

Within this study, we demonstrated that the levels of KIM-1 in the sera of FMF patients were statistically significantly higher compared to healthy controls (p=0.040). However, we did not observe a significant relationship between urinary protein/creatinine ratio, eGFR levels, acute phase reactants, and s-KIM-1 levels in the FMF group. Additionally, we showed that serum sKIM-1 levels were significantly lower in patients using canakinumab in the FMF group compared to those who did not.

Early detection of kidney damage due to FMF may benefit from non-invasive biomarkers that reflect disease-related renal involvement. Currently, the best way to demonstrate FMF-related kidney damage is still through kidney biopsy. Therefore, the use of biomarkers in patients where kidney biopsy is not feasible may be beneficial. It is well known that KIM-1 is a biomarker for the early diagnosis of kidney damage, predicting the severity of damage, and its outcomes.^34^

One of the initial manifestations of kidney amyloid damage due to FMF is proteinuria.^35^ However, in our study, although urinary protein/creatinine ratio (UPCR) was higher in the FMF patient group compared to the control group, no statistically significant relationship was found between the two groups. It has been traditionally thought that proteinuria in FMF is solely attributed to amyloidosis; however, besides amyloidosis, other nephropathies have been identified. In a study, FMF patients with non-amyloidosis proteinuria underwent kidney biopsy, revealing that 60% of 25 patients were diagnosed with amyloid kidney disease (AKD) and 40% with non-amyloid nephropathy.^36^ As observed in this study, proteinuria may not be detected in the early stages. Therefore, there is a need for non-invasive markers capable of detecting early-stage damage. In a study conducted as a result of this need, neutrophil gelatinase-associated lipocalin (NGAL) was found to be elevated in FMF patients.^37^ However, to the best of our knowledge, there have been no reports of a study regarding KIM-1 levels in FMF patients.

The high levels of KIM-1 in the FMF patients group support the notion that FMF can cause kidney damage through mechanisms other than amyloidosis. It is highly likely that this mechanism is related to inflammation. Associations of FMF with inflammatory diseases such as glomerulonephritis, rheumatoid arthritis, and Crohn’s disease have been described in the literature.^38–40^ In our study, the significant decrease in KIM-1 levels in FMF patients using canakinumab supports this perspective. However, we did not observe a significant relationship between KIM-1 and patients using colchicine or anakinra in our study.

## LIMITATIONS

This study has several limitations, the lack of simultaneous measurement of serum and urine KIM-1 levels, and its cross-sectional design. The small number of samples in the subgroups may have affected the ability to perform sound statistical analysis. Although there is no statistically significant difference, the difference in mean age between the groups may have possible effects on KIM-1 levels. In particular, the significantly lower number of patients using canakinumab (n: 4) compared to patients not using it (n: 46) may have prevented a sound statistical analysis in the comparison in terms of KIM-1. However, this study is the first to demonstrate that s-KIM-1 levels are statistically significantly higher in FMF patients compared to healthy controls and that sKIM-1 levels are significantly lower in patients using canakinumab compared to those who do not.

## CONCLUSION

Serum KIM-1 levels were found to be higher in FMF patients compared to healthy controls, and individuals receiving canakinumab showed significantly lower levels. These findings suggest that KIM-1 might have potential as a non-invasive biomarker for monitoring subclinical kidney involvement and treatment response in FMF, particularly in the context of IL-1 inhibition. However, considering the similar levels of traditional renal markers such as creatinine, urea, and only marginal differences in UPCR, the clinical utility of KIM-1 as a monitoring tool remains to be clarified. Further prospective and mechanistic studies are warranted to better define its role in FMF pathophysiology and its applicability in therapeutic contexts.

## Data Availability

Not applicable.
